# Deep neural models for color classification and color constancy

**DOI:** 10.1167/jov.22.4.17

**Published:** 2022-03-30

**Authors:** Alban Flachot, Arash Akbarinia, Heiko H. Schütt, Roland W. Fleming, Felix A. Wichmann, Karl R. Gegenfurtner

**Affiliations:** 1 Abteilung Allgemeine Psychologie, Justus Liebig University, Giessen, Germany; 2 Center for Neural Science, New York University, New York, NY, USA; 3 Experimental Psychology, Justus Liebig University, Giessen, Germany; 4 Neural Information Processing Group, University of Tübingen, Germany

**Keywords:** color constancy, deep learning, spectral renderings, color classification

## Abstract

Color constancy is our ability to perceive constant colors across varying illuminations. Here, we trained deep neural networks to be color constant and evaluated their performance with varying cues. Inputs to the networks consisted of two-dimensional images of simulated cone excitations derived from three-dimensional (3D) rendered scenes of 2,115 different 3D shapes, with spectral reflectances of 1,600 different Munsell chips, illuminated under 278 different natural illuminations. The models were trained to classify the reflectance of the objects. Testing was done with four new illuminations with equally spaced CIEL*a*b* chromaticities, two along the daylight locus and two orthogonal to it. High levels of color constancy were achieved with different deep neural networks, and constancy was higher along the daylight locus. When gradually removing cues from the scene, constancy decreased. Both ResNets and classical ConvNets of varying degrees of complexity performed well. However, DeepCC, our simplest sequential convolutional network, represented colors along the three color dimensions of human color vision, while ResNets showed a more complex representation.

## Introduction

Color constancy denotes the ability to perceive constant colors, even though variations in illumination change the spectrum of the light entering the eye. Although extensively studied (see Gegenfurtner & Kiper, 2003; Witzel & Gegenfurtner, 2018; Foster, 2011, for reviews), it has yet to be fully understood. Behavioral studies disagree on the degree of color constancy exhibited by human observers (Witzel & Gegenfurtner, 2018), and color constancy is considered an ill-posed problem. It is argued from theoretical and mathematical considerations that perfect color constancy is not possible using only the available visual information (Maloney & Wandell, 1986; Logvinenko et al., 2015). Yet, observing that humans do achieve at least partial color constancy sparks the question about which cues and computations they use to do so. It also remains unclear which neural mechanisms contribute to color constancy. Low-level, feedforward processes, such as adaptation and the double opponency of cells in early stages of the visual system, have been identified as being useful for color constancy (Gao et al., 2015). Yet, other studies suggest that higher-level and even cognitive processes such as memory also contribute. For example, better color constancy has been observed for known objects than for unknown ones (Granzier & Gegenfurtner, 2012; Olkkonen et al., 2008). Thus, we are still lacking a complete neural model of color constancy, which encompasses physiological similarities to the primate's visual system and at the same time exhibits similar behavior to humans on color constancy relevant tasks.

In contrast to earlier computer vision approaches, deep neural networks (DNNs) may have greater potential to be models for biological color constancy and color vision. Conceptually inspired by biology (LeCun & Bengio, 1995), DNNs can solve many complex visual tasks such as face and object recognition (Zeiler & Fergus, 2014; Yosinski et al., 2015), and DNNs trained for object recognition have been shown to correlate with neuronal activity in visual cortical regions (Güçlü & van Gerven, 2015; Cichy et al., 2016). The predictions for cortical activity are not perfect, though, and DNN responses are far less robust to distortions of the input images than human observers (Goodfellow et al., 2014; Brendel et al., 2017; Geirhos et al., 2017, 2018; Akbarinia & Gil-Rodríguez, 2020). Furthermore, it has been shown that current DNNs and human observers do not agree which individual images are easy or difficult to recognize (Geirhos et al., 2020b).

For the processing of color information specifically, similarities have been observed between DNNs trained on complex tasks and the visual system (Rafegas & Vanrell, 2018; Flachot & Gegenfurtner, 2018). In addition, DNNs trained on illumination estimation from images have outperformed all previous approaches (Lou et al., 2015; Bianco et al., 2015; Hu et al., 2017; Shi et al., 2016; Afifi & Brown, 2019). This success was enabled by fine-tuning networks pretrained on other tasks (Lou et al., 2015), various data augmentation techniques including the application of additional color distortions and cropping (Lou et al., 2015; Bianco et al., 2015), and architectural innovations and adversarial training (Hu et al., 2017; Shi et al., 2016; Afifi & Brown, 2019). Notably, none of these networks were trained only on natural variation in illuminations, and most of them aimed at the task of color-correcting images, not estimating object color.

Color constancy is also a well-studied problem in computer vision and image processing, yet the extent to which the algorithms in these engineering fields can inform our understanding of human color constancy is limited. In those fields, color constancy is typically approached by explicit estimation of the scene's illumination (Land, 1964; Akbarinia & Parraga, 2017; Afifi & Brown, 2019; Bianco & Cusano, 2019; Hu et al., 2017), followed by an image correction via the von Kries assumption (von Kries, 1902). In biological vision, however, color constancy is rather tested as the ability to extract color information about the object and materials in the scene consistently across varying illuminations (Maloney & Wandell, 1986; Foster, 2011; Witzel & Gegenfurtner, 2018; Weiss et al., 2017; Olkkonen et al., 2008), thus going one step further than illumination estimation and requiring some form of color comprehension.

Deep learning approaches to color constancy are limited by their need for large datasets. The heavy requirements for a good color constancy image dataset (calibrated cameras, pictures taken from the same angle at different times of day, or with many different controlled and measured illuminations) result in datasets rarely containing more than a thousand images.^1^ One approach to generate larger training datasets for this kind of situation is to use computer graphics to render images or videos instead. This approach has successfully been used for depth and optical flow estimation tasks (Butler et al., 2012; Dosovitskiy et al., 2015; Ilg et al., 2018), as well as other aspects of surface material inference, such as gloss perception (Storrs et al., 2021; Prokott et al., in press), but has to our knowledge not been applied to color constancy yet.

The goal of this study is (1) to teach DNNs to identify color in settings that require color constancy, (2) to assess whether the trained models exhibit behaviors akin to observations made in psychophysical studies for color constancy, and (3) to test whether human-like color representations emerge with training. To do so, we proceeded as follows: We generated artificial training and validation images using three-dimensional (3D) spectral rendering with a naturalistic distribution of illuminations to overcome the limitations of previous approaches. Instead of RGB encoded inputs, we used images encoded using human cone sensitivities. Instead of training our models on illumination estimation, we trained them to extract the color of a foreground object within the scene. Specifically, the task was to classify objects floating in a room based on their surface color, under a large set of different illumination conditions. Chromaticities of colored surfaces and illuminations were such that color constancy was necessary to attain high accuracy, that is, the chromaticity shifts induced by colorful illuminations were often larger than the chromaticity difference between neighboring surfaces. We then devised an evaluation procedure of the trained models to allow comparison with human studies. Finally, instead of using only a large, complicated standard deep learning model, we trained both complex and relatively simple ones and compared their performance as well as the color representations they developed during training.

We found that all our models performed very well at recognizing objects surface colors, even for illuminations they had never seen, with a supra-human accuracy. Like humans (Kraft & Brainard, 1999), the accuracy of the models drastically degraded, however, as we manipulated the input by gradually removing cues necessary for color constancy. Similarly, we also found a better performance for illuminations falling along the daylight axis than for illuminations falling in the orthogonal direction. This result is in line with observations made in psychophysical studies (Pearce et al., 2014; Aston et al., 2019). We found, however, that different architectures learned to represent the surface colors of objects very differently. One of them, *DeepCC*—the most straightforward convolutional architecture we implemented—seems to represent surface colors following criteria resembling the perceptual color dimensions of humans, as determined by psychophysical studies. Other architectures like ResNets, on the other hand, did not. This suggests that while perceptual color spaces may aid color constancy, they are certainly not necessary for achieving human-like robustness to changes in illumination.

This article is divided into sections following our main findings. We start by reporting the results obtained for DeepCC's evaluation, with a focus on the effect of illumination on DeepCC's performance. Then we analyze how DeepCC represents surface colors and gradually becomes color constant throughout its processing stages. We finish with a summary of the results obtained for other deep net architectures, in particular, custom ResNet architectures.

## General methods

### Munsell and CIEL*a*b* coordinates

Throughout this study, two-color coordinate systems are used. The first one is the Munsell color system (Munsell, 1912; Nickerson, 1940), defined by the Munsell chips themselves. Each Munsell chip is indexed according to three coordinates: *Hue*, *Value*, and *Chroma*. *Hue* is divided into 5 main hues: Red, Yellow, Green, Blue, and Purple, each one divided into 8 intermediary hues, for a total of 40 hues. *Value* is close to *lightness* as it refers to how light a Munsell chip is perceived to be. In terms of surface reflectance, it approximately corresponds to the amount of light that gets reflected by the Munsell chip, that is, the area under curve (Flachot, 2019). *Value* varies from 0 to 10, 0 being the darkest and 10 being the lightest. *Chroma* refers to the colorfulness of the chip, or its distance from gray. In terms of surface reflectance, it corresponds to the contrast in the amount of light reflected by different wavelengths. The higher the chroma, the less flat the surface reflectance spectrum (Flachot, 2019) and the more colorful the chip. *Chroma* varies from 0 to 16. Note, however, that the Munsell color system does not have perfect cylindrical shape but has a limited gamut: Certain hues and values do not allow for high chromas. Hence, the full set of Munsell chips consists of only 1600 chips instead of 40×16×10=5,600 chips. Because the Munsell color system is defined by the Munsell chips, it is the most appropriate space to discriminate Munsells. In addition, the Munsell chips were chosen in an attempt to be perceptually uniformly distant, and as such, the Munsell coordinate system is an approximately perceptually uniform space.

Another perceptually uniform color space is the CIEL*a*b* (Ohno, 2000) coordinate system. It was constructed such that its Euclidean distance, commonly called ΔE, is an approximate measure of perceptual difference: Two colors equidistant to another in CIEL*a*b* are *approximately* perceptually equidistant. Additionally, it is commonly considered that the average just noticeable difference (JND) between two colors is approximately 2.3 ΔE (Mokrzycki & Tatol, 2011), meaning that a human observer is not able to discriminate two color patches closer than this value, even if placed side-by-side. Of the three dimensions, L* accounts for lightness, a* accounts for greenish-reddish variations, and b* accounts for blueish-yellowish variations. The white point (point of highest Lightness) was computed using the spectrum of the light reflected by the Munsell chip of highest value, under the D65 illumination. This Munsell chip is also an achromatic chip.

To relate the two color coordinate systems, the median distance between two adjacent Munsell chips is equal to 7.3 ΔE (i.e., significantly above the JND).

### Image generation

**Figure 1.**
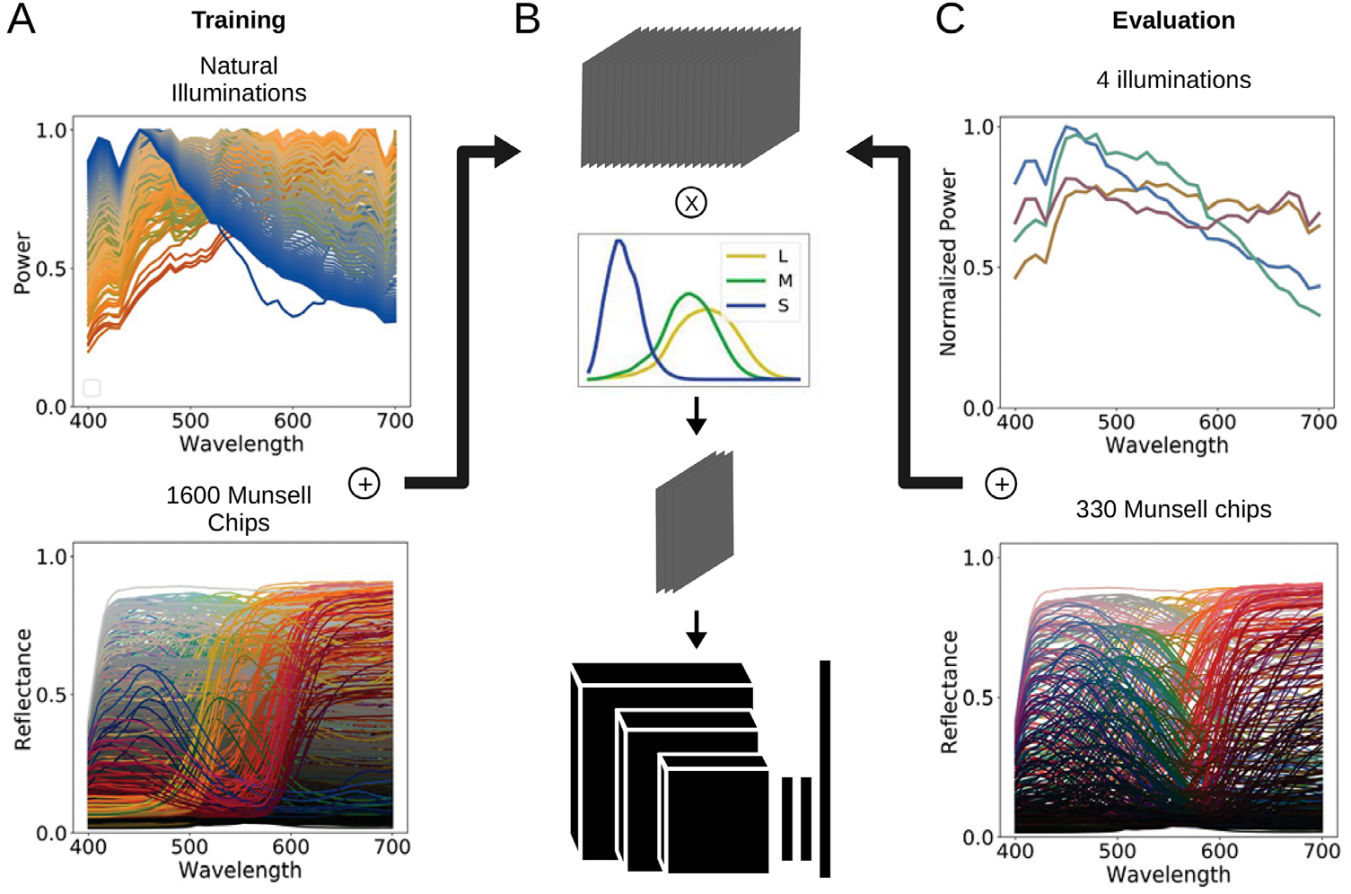
Figure illustrating our method, both for training and evaluation. (A) To generate the training set of images, sets of 279 spectra of natural illuminations and 1,600 spectra of Munsell reflectances were used. The resulting multispectral images (B) were then converted into three “LMS” channels using human cone sensitivity spectra and fed to the network. (C) The four illuminations R, G, Y, and B were used exclusively in the evaluation. Note that while Y and B fall on the daylight locus, R and G have chromaticities different from the illuminations of the training set. Out of 1,600, only 330 Munsell spectra were used.

In the present study, we generated our own images using the physically based renderer.^2^ Mitsuba was developed for research in physics and includes accurate, physics-based approximations for the interaction of light with surfaces (Pharr et al., 2016; Bergmann et al., 2016), yielding a perceptually accurate rendering (Guarnera et al., 2018). Most important, it also allows the use and rendering of spectral data: One can use physically measured spectra of lights and surfaces as parameters. Outputs can also be multispectral images rather than simple RGB images. We exploited this multispectral characteristic of Mitsuba using the reflectance spectra of 1,600 Munsell chips (Munsell, 1912) downloaded from Joensuu University^3^ (Kalenova et al., 2005). As illuminations, we used the power spectra of 279 natural lights: 43 were generated from the D series of CIE standard illuminations (Judd et al., 1964; Ohno, 2000) at temperatures ranging from 4.000K to 12.000K; 236 were taken from the forest illuminations measured by (Chiao et al., 2000). Each illumination spectrum was normalized such that their highest point reaches the same, arbitrary value of a 100. The spectra of both Munsell reflectances and illuminations are displayed in Figure 1A.

For meshes, we used a compilation of object datasets issued by Evermotion^4^ for a total of 2,115 different meshes, ranging from human-made objects to natural objects. Each mesh was normalized such that they have the same size (equal longest dimension).

In order to approximate the input to human visual processing, we first generated our images with 20 channels, at equally spaced wavelengths ranging from 380 to 830 nm. These were then collapsed onto three “LMS” channels using measured human cone sensitivities (Stockman & Sharpe, 2000). Images were saved with floating points, thus without the need for any gamut correction or further processing. This procedure is illustrated in Figure 1B.

**Figure 2.**
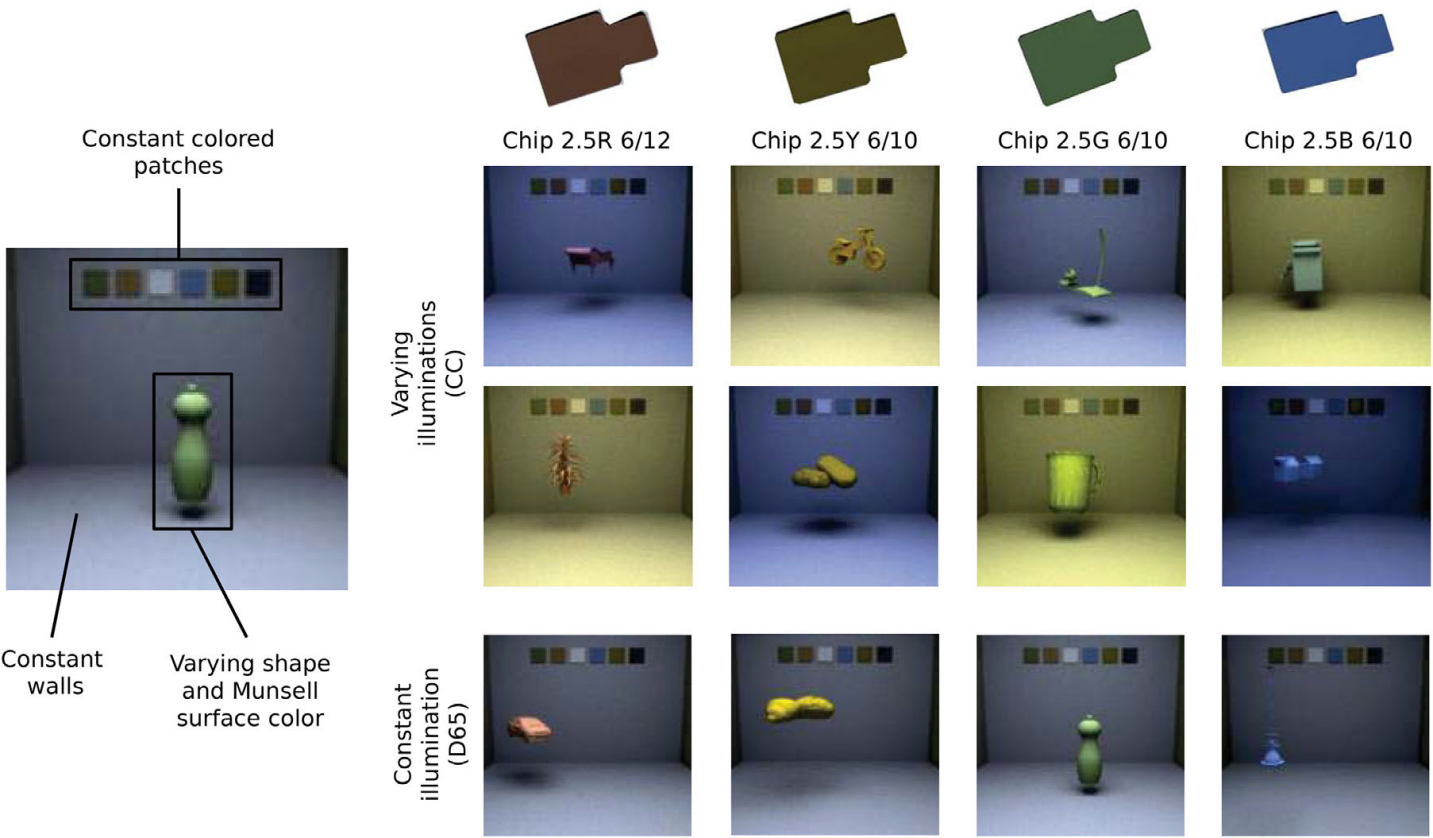
Illustration of the two training datasets used: one with varying illumination (CC), another with a constant illumination (D65). The classification task consisted of identifying the correct Munsell chip used as surface reflectance for a random object floating in the room. In order to be performant on the CC dataset, the network had to account for the illumination.

The 3D scene consisted of a simple “room” (see Figure 2), with three walls, a floor, and a ceiling with constant Munsell reflectances as surfaces. On the ceiling, a rectangular light source was defined. On the back wall, six colorful patches with constant Munsell reflectances were added. Their purpose was giving additional cues for the model to solve color constancy, as seems to be necessary for humans (Brainard et al., 2003; Yang & Maloney, 2001).

Finally, each LMS image consisted of a random object floating at a random position and orientation in the scene, with a given Munsell surface reflectance. The shape of the object was taken randomly among our pool of 2,115 meshes. Although its position was also random, it was bounded so that the object would never occlude the six patches in the background and would stay fully within the field of view. We generated two datasets, the *Set-CC* and *Set-D65* datasets. Illustrations of these datasets are available in Figure 2. In the *CC* dataset, we generated 279 images per Munsell chip, one for each of the 279 natural illuminations. In the *D65* dataset, we also generated 279 images per Munsell chip value but kept the illumination constant with the power spectrum of the standard D65 illumination. Each dataset thus consisted of 1,600×279=446,400 images, with a resolution of 128×128 pixels and three color channels, one for each L, M, and S cone photoreceptor. Images were labeled according to the mesh type, object position, illumination, and, most important in this study, according to the Munsell chip used for the mesh's surface reflectance. All surfaces were defined as Lambertian. This dataset is publicly available,^5^\as well as the pipeline to generate it.

### Deep architecture

One network architecture has been extensively studied throughout this work. Several others were also tested, evaluated, and analyzed, for which results are described in detail in “Standard and custom architectures.” For now, we limit ourselves to describing the network architecture most relevant for this study, which we refer to as *Deep*.

*Deep* has a convolutional architecture (LeCun et al., 1998; Krizhevsky et al., 2012) with three convolutional layers and two fully connected layers preceding a classification layer. Convolutional layers can be described as a set of linear kernels. Each kernel applies the same linear filter of limited size on different portions of the input, at regular intervals. The output of one linear filter applied on one input patch, coupled with a half-wave rectification (ReLU), is the output of one unit. Units in convolutional layers have thus limited receptive fields in the image input. Fully connected layers instead take all units of the previous layer as input, such that the units’ receptive fields cover the whole input image. The convolutional layers of the *Deep* architecture have 16, 32, and 64 kernels with kernel sizes of 5, 3, and 3, respectively. After each convolutional layer follows a 2×2 maxpooling layer. The fully connected layers have 250 units each. The classification layer is a simple fully connected layer, preceded by a 40% dropout layer for regularization.

*Deep*'s input consisted of the set of images we generated, thus with a dimension of 128×128 pixels and three color channels, one for each L, M, and S cone photoreceptor.

### Task and training

The training was supervised with the learning objective of outputting the Munsell chip label for each image (i.e., the color of the object in each scene). Cross-entropy was used as loss. Training took place for 90 epochs. We used the Adam optimizer (Kingma & Ba, 2015), with a learning rate of 0.001, divided every 30 epochs by 10.

We trained separate models on the *CC* and *D65* datasets. Each dataset was further divided into training and validation subsets, the former consisting of 90% of the dataset's images and the latter the remaining 10%. Training and validation subsets are quite similar: They use the same viewpoint and the same room, although the floating object was at random position and orientations. But they also have differences: They do not use the same object meshes, and in the case of *CC*, neither do they use the same illumination spectra. The validation subsets were generated with 212 object meshes and for *CC* 28 illumination spectra exclusively, selected randomly among the 2,115 meshes and 279 illuminations. The remaining meshes and spectra were used for generating the training subsets. Training subsets were only used for training our models, while the validation sets were only used for testing the model during training at regular intervals (each epoch) to monitor its performance on images it had never seen.

We can now see how our task requires the models to become color constant: In order for the models to achieve a high recognition accuracy on the *CC* dataset, they would need to compensate for the chromatic shifts that are induced by the varying illuminations interacting with the Lambertian surfaces. By extension, this means they would need to achieve some degree of color constancy. Indeed, the standard deviation of the training illumination's distribution is equal to 8.55 ΔE, higher than the median distance between two adjacent Munsell classes of 7.3 ΔE. Out of the 279 illuminations in our training and validation sets, 202 are distant by more than 10 ΔE from the reference illumination D65.

Given that there are two datasets, *CC* and *D65*, two kinds of training instances need to be distinguished: *DeepCC* when trained on *CC* and *Deep65* when trained on *D65*. Due to several randomization procedures implemented during training, two training instances of the same architecture trained on the same dataset will give slightly different results. To allow broader claims and a statistical analysis, we trained 10 instances of *DeepCC* and *Deep65* each.

Each model was trained on one GeForce GTX 1080. Batch size varied from architecture to architecture but was maximized to fit the GPUs memory. In the case of *Deep*, the batch size was 800 images. All the code is available on Github.^6^

Other than the validation dataset, we devised other datasets to further evaluate our models. These evaluation datasets mimicked the typical experimental procedures for studying color constancy, consisting in removing or ambiguously modifying contextual cues to make the task more difficult (Witzel & Gegenfurtner, 2018; Kraft et al., 2002). They facilitated identifying the relevance of diverse cues for the task, the testing the model's robustness to scene modifications, and the comparison with previous psychophysical studies. These contextual modifications were (1) removing the colored patches in the background—if the models use the constancy information transmitted by these patches, a drop in performance should follow. (2) Swapping the colored patches in the background with patches under a different illumination—again, if the models use the constancy information transmitted by these patches, a drop in performance should follow. (3) Placing the floating object in a background illuminated with a wrong illumination—if the models follow the information within the scene to estimate the illumination's color, then the resulting incorrect estimation should lead to a misclassification of the floating object's color.

A detailed description of the evaluation datasets will follow in “Evaluation DeepCC and Deep65” and “Impoverished visual scene,” sections where the results of these evaluations are presented.

### Metrics

To assess the performance of *DeepCC* and *Deep65*, we used several measures of accuracy. Given that the task is the classification of Munsell chips, two are the standard top-1 and top-5 accuracies (Krizhevsky et al., 2012): top-1 counts as hit when the correct Munsell is the one selected as most probable by the model; top-5 counts as hit when the correct Munsell is among the five selected as most probable by the model. In addition, we defined the Muns${}^{3}$ accuracy: A hit occurs whenever the Munsell selected as most probable by the model is 1 Munsell away from the correct one (within a cube of side 3 in Munsell space centered on the correct Munsell).

Due to their discrete nature, however, top-1, top-5, and Muns${}^{3}$ accuracies do not discriminate between cases when a model selected a Munsell just outside Muns${}^{3}$ or when it was completely off. To correct this shortcoming, we converted the model's output into chromaticity coordinates. We did so by considering the Munsell chips’ chromaticities under the D65 illuminant in CIEL*a*b* space. We then defined the model's *selected chromaticity* as the chromaticity of the Munsell selected by the model. The Euclidean distance between the correct Munsell's chromaticity and the model's selected chromaticity now defines a continuous measure of the model's error. Following the literature (Ohno, 2000; Weiss et al., 2017), we call this error ΔE (with its 1976 definition).

To further compare with the color constancy literature, we considered another measure called the *Color Constancy Index* (CCI) (Foster, 2011; Arend & Reeves, 1986; Weiss et al., 2017). This measure has the benefit of taking into account the quantitative error of the model in color space (ΔE) relative to chromaticity shift induced by the illumination. Consider that we present to the model an image showing a floating object under an illumination I with the surface reflectance of a Munsell M. Consider now that the model recognizes the wrong Munsell N. Then the Color Constancy Index is defined as

(1) $$\begin{array}{ccc} CCI\; & = & 1-\frac{\left|C_{I}^{N}-C_{I}^{M}\right|}{\left|C_{D65}^{M}-C_{I}^{M}\right|},\\ & = & 1-\frac{\Delta E}{\left|C_{D65}^{M}-C_{I}^{M}\right|}.\; \end{array}$$

where $C_{I}^{M}$ is the chromaticity of the Munsell M under the illumination *I*, $C_{D65}^{M}$ is the chromaticity of the same Munsell chip but under the standard illumination D65, and $C_{I}^{N}$ is the chromaticity of Munsell N under the illumination I and recognized by the model. If the model recognizes the correct Munsell, then the ratio in the formula is neutral and CCI would be equal to 1. However, if the model does not compensate for the illumination's shift in chromaticity and recognizes the wrong Munsell chip, CCI would be close to 0. Negative values of CCI indicate that the network chose the wrong Munsell for other reasons, beyond the chromaticity shifts induced by the illumination.

## DeepCC and Deep65 evaluation

This section focuses on the evaluation of DeepCC and Deep65. Results for other architectures can be found in “Standard and custom architectures.”

We first present the results of training and validation for both DeepCC's and Deep65's instances. We then present thorough evaluations of the models using additional, custom datasets (description below).

We found that both DeepCC and Deep65 reached high top-1 accuracies on their respective validation datasets. DeepCC instances reached on average 76% accuracy on the CC validation set, while Deep65 reached on average 86% accuracy on the D65 validation set. These values clearly show that the two sets of networks learned how to solve their task and are able to differentiate between 1,600 different surface colors reasonably accurately (random performance would be 0.0625%). The higher performance of the Deep65 network also indicates, as expected, that the D65 task is inherently easier than when illumination is allowed to vary, and thus color constancy is required to perform the task.

In order to evaluate DeepCC in greater detail, as well as allowing some comparison with observations made in psychophysical studies, we generated another set of testing images, with settings closer to conditions found in typical perceptual experiments.

### Methods

To facilitate our analysis, an evaluation dataset was generated using a slightly different procedure than for the training sets. First, a subset of 330 Munsell chips was used, instead of the original set of 1,600 (cf. Figure 1C). This subset was originally used for the World Color Survey and is now a standard for studies focusing on color naming (Berlin & Kay, 1969). It is also widely used in studies related to color categories (Witzel, 2019) and unique hues (Philipona & O’Regan, 2006; Flachot et al., 2016). As such, they are an excellent basis for comparing our models with human judgments.

Second, we used four illuminations (cf. Figure 1C) equidistant to the CIEL*a*b* gray point by 10 ΔE (Ohno, 2000) in the chromaticity plane. This procedure was inspired by experimental studies on illumination discrimination and estimation (Aston et al., 2019). Two, B and Y, lie on the daylight locus projected onto the chromatic plane, and are thus within the distribution of the natural illuminations used during training. The other two, G and R, lie in the orthogonal direction, which crosses the daylight locus at the gray point, and are outside of the distribution of illuminations used during training. More precisely, G is 4.45 ΔE away from its closest illumination within the training set, while R is 7.9 ΔE away, making R then G the two illuminations DeepCC is less familiar with. Their power spectra were generated with the principal components of natural daylight spectra defined by Judd et al. (1964), which serve as the basis for the D series of the CIE standard illuminations. These illuminations were normalized such that their areas under curve were equalized, thus minimizing their difference in Lightness. For each Munsell of the 330 Munsell classes and each of the four illuminations, we generated 10 images for a total of 330×4×10=13,200 images.

Note the fundamental difference between the validation sets employed earlier and the evaluation set defined here: While the validation datasets consisted of illuminations and 3D shapes the networks had never seen (to prevent overfitting), these illuminations and shapes were still taken randomly from the same distributions as for the training set (see General methods). The evaluation dataset, however, included illuminations that were completely outside of the illumination distribution used at training time. As such, our evaluation procedure is in accordance with the recommendations from the machine learning community and formally defined recently (Geirhos et al., 2020a): using one *independent and identically distributed* (i.i.d.) test set—our validation set—and another *out of the distribution* (o.o.d.) test set—the evaluation set described here.

Although the illumination spectra were different from the ones used during training and validation, the scene in which the floating objects were displayed was exactly the same. We therefore refer to this evaluation dataset as *normal*. Because we are evaluating *DeepCC* and *Deep65*, each trained on different datasets, we distinguish between two conditions: *CC* and *D65*.

### Results

**Figure 3.**
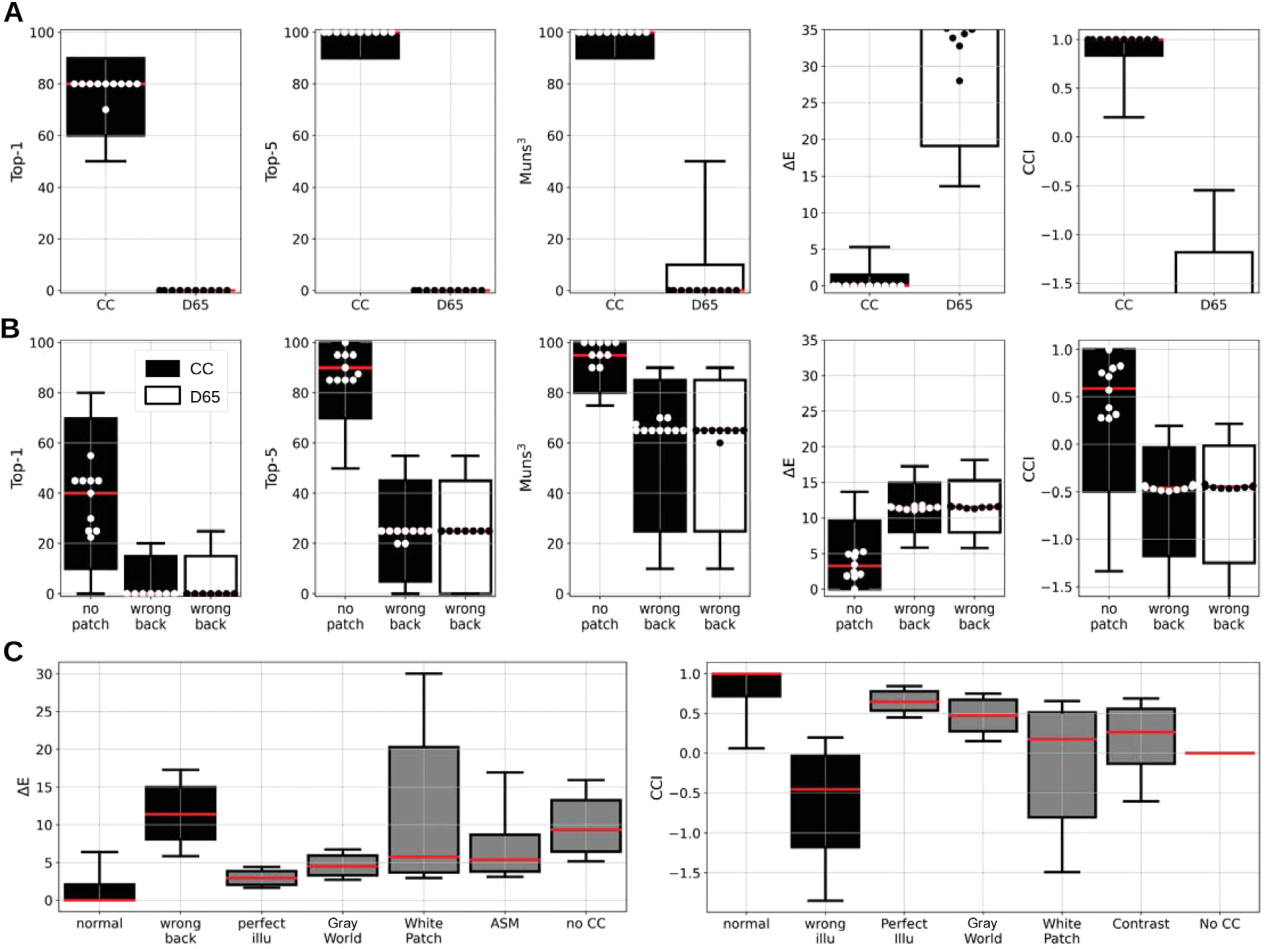
DeepCC's evaluation results obtained for all measures and all conditions. Each column corresponds to one measure (*Top-1*, *Top-5*, and *Muns*${}^{3}$ accuracies, ΔE errors to ground truth, and CCI). Boxplots show distributions across Munsell chips; the swarm shows the performance of the 10 training instances. (A) Performance for models trained under varying illuminations *CC* or the D65 illumination only *D65* (“DeepCC and Deep65 evaluation”). The models trained on the *CC* dataset learned to classify Munsell colors accurately even under novel illuminations, contrary to the models trained on *D65* only. (B) In black, performance of DeepCC under the *no patch* and *wrong back* conditions (“Impoverished visual scenes”). In white, performance of Deep65 under the *wrong back* condition. DeepCC learned to rely on contextual cues within the scene to solve the task. When these cues are taken away or incongruously altered, the model's performance decreases. Under the wrong back condition, where the background is artificially kept constant despite various illuminants shining on the object, DeepCC performs at the level of Deep65. (C) Performance of DeepCC compared to other approaches to color constancy (“Classical approaches”), namely, perfect illumination estimation and von Kries adaptation (perfect illu), Gray World, White Patch, ASM, and no account for illumination whatsoever (no CC). Under the normal condition, DeepCC performed better than any algorithm tested, even better than a model that would perfectly estimate the illumination and then perform the standard von Kries adaptation (perfect illu condition).

Figure 3A shows the distributions obtained for each of our five metrics under the CC and D65 conditions. For the accuracies, we considered the distributions of values found for each Munsell class and illuminations (each point of the distribution is thus computed with 10 images). For ΔE and CCI, we plot the distributions of values found for individual images. Under the CC condition, we found median top-1, top-5, and Muns${}^{3}$ accuracies of 80%, 100%, and 100%, respectively, across Munsell classes. The first quartiles are at 60%, 90%, and 90%, respectively. This means that for the majority of Munsell classes, DeepCC selects the correct Munsell class in four out of five images, and when wrong, it still selects a neighboring chip. This is confirmed by the distributions found for ΔE and CCI, with median values of 0 and 1. Eighty-five percent of the images yielded less than 5 ΔE error as indicated by the whiskers, 93% less than 10 ΔE error, and 99% less than 19 ΔE. As a comparison, note that the median ΔE distance between adjacent chips is approximately 7.5. This means that when DeepCC instances selected the wrong chip, it tended to be a close neighbor of the correct one. This is confirmed by the Muns${}^{3}$ accuracy, according to which the model had an accuracy equal to or above 90% for 95% of the Munsell classes. Similarly, DeepCC showed a CCI higher than 0.83 in 75% of cases. This CCI value of 0.83 is among the higher end of CCI values measured in humans psychophysical experiments (cf. Foster, 2011; Witzel & Gegenfurtner, 2018, for reviews), thus indicating the supra-human performance of the model on this dataset. We also found a positive CCI value in more than 87% of cases, evidence that DeepCC not only learned to discriminate between Munsell colors with high accuracy but also learned to account for potential color shifts induced by the illumination.

Results were, however, very different for Deep65—the network trained using only a single illuminant, D65. We found median values of 0 in all three accuracies, meaning the 10 training instances of Deep65 rarely came close to selecting the right Munsell class. This is made clear with the distributions of the ΔE and CCI measures. For the vast majority of the images, Deep65 exhibited errors of above 10 ΔE and negative CCI, meaning that Deep65's error cannot be explained by the illumination change alone. This indicates that Deep65 lacks the ability to cope with illuminant deviations from the one it has been trained on, whereas DeepCC could generalize to novel illuminants beyond the 279 different illuminants it had been trained upon.

### Interim conclusion

Results so far show that DeepCC did learn to accurately classify color surfaces under varying illumination. In doing so, it also learned to discount the illumination color, reaching a high degree of color constancy, even for illuminations outside of the gamut of illumination spectra used for training. Deep65, on the other end, performed very poorly on the four illuminations used for testing.

## Impoverished visual scenes

We have seen that DeepCC achieved supra-human performance under normal conditions on the devised evaluation dataset, thus achieving some degree of color constancy. A remaining question is which elements within the scene DeepCC used to compensate for illumination change: Does it consider, for example, the six constant color patches in the background? Given that there are interreflections between the floating object and the surrounding walls, is there any need for the model to use cues in the background at all?

Computer graphics allow us to manipulate the scene elements to test these questions. We thus devised new datasets to gain insights into which cues within the images DeepCC might use to achieve color constancy. Three manipulations were conducted: (1) removing the constant patches in the background, (2) modifying the colored patches in the background to have the wrong color, and (3) showing a floating object illuminated by one illumination in a scene illuminated by another illumination.

We then tested DeepCC on these three new datasets, without any additional training.

### Methods

**Figure 4.**
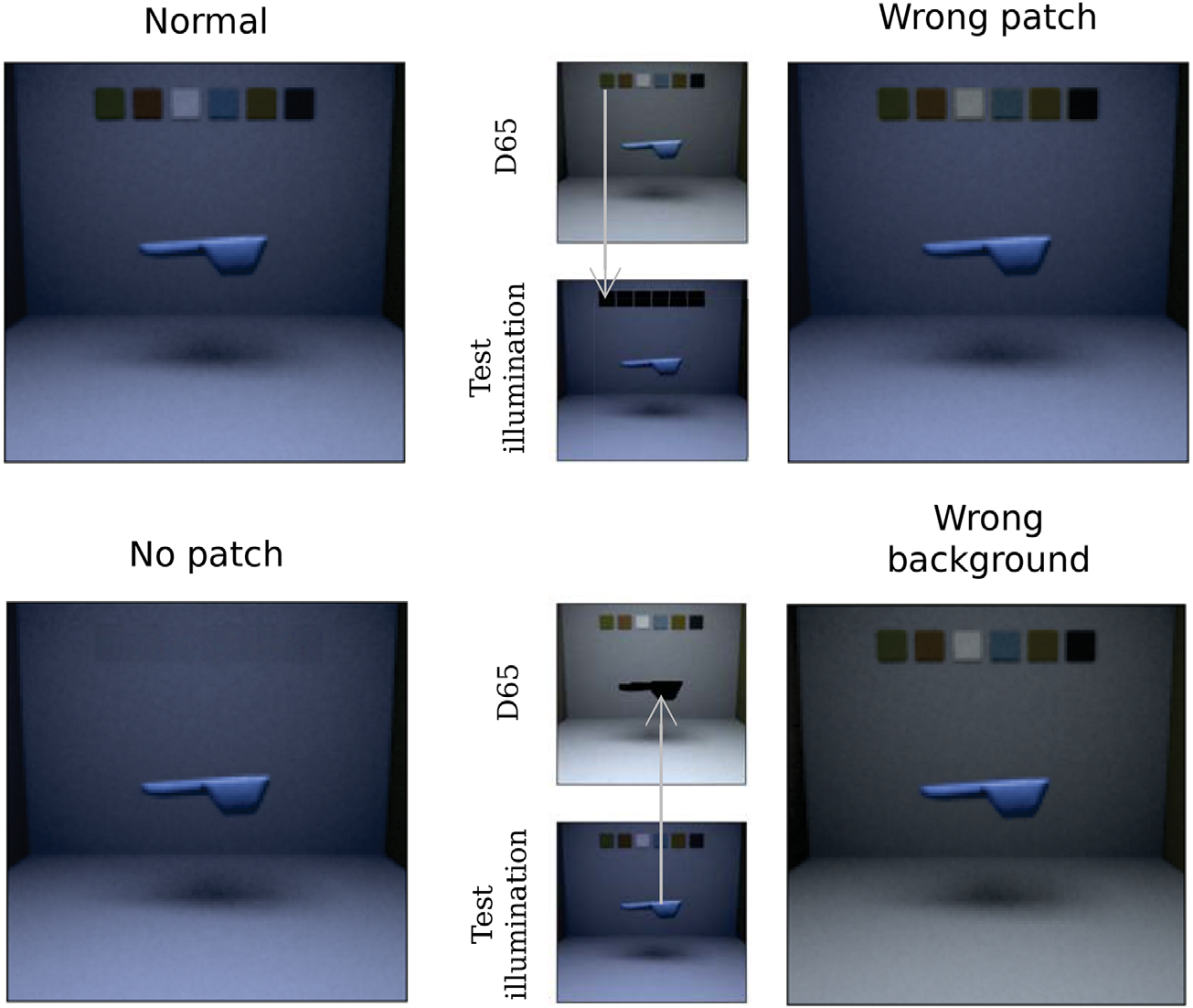
Example for the four types of images we used during testing: normal, no patch (the colored patches in the background are removed), wrong patch (the colored patches are cropped and replaced by the same surfaces but under the D65 illumination), and wrong background (the object in cropped and placed in the room illuminated by D65 illumination).

We generated three new image datasets to test DeepCC, in which some elements within the scene were removed or incongruously modified. These elements constituted cues that are known to be useful to humans for achieving color constancy. Previous experiments (Kraft et al., 2002) have shown that increasing the color cues within a scene, in their case adding a Macbeth color checker, can increase color constancy for humans. Thus, in one dataset, the *no patch* dataset, we removed the six constant patches located on the back wall. If the networks do partially rely on the information given by the background patches to solve color constancy, then the missing information should lead to a drop in model performance. Other studies (Kraft & Brainard, 1999) showed that human color constancy is neutralized when the context surrounding the object of interest is manipulated incongruously. Thus, in two other datasets, *wrong patch* and *wrong background*, we gave the network conflicting contextual cues. In *wrong patch*, we modified the chromaticities of the six colored patches, originally under one of the four test illuminations, to be replaced by their color under the D65 illumination. In *wrong background*, the floating object, illuminated by one of the four test illuminations, was cropped out and placed in the same scene but illuminated by the D65 illumination. If the networks do use the background information to solve color constancy, then the misleading information should also lead the models’ performance to drop, and significantly more so than in the *no patch* condition. Note that for the last condition, human observers would be expected to be unable to solve the task. Examples of images illustrating these conditions are shown in Figure 4.

### Results

Results are shown in Figure 3B. The results for DeepCC are plotted in black and the results for Deep65 under the *wrong background* condition are plotted in white. Overall, DeepCC performed significantly worse in each of the three new conditions than in the normal condition, but still better than Deep65 in the normal condition. Performance for the *no patch* condition was on average still fairly high, indicating that the networks did not rely solely on the constant patches to perform the task. The three accuracy distributions include medians of 40%, 90%, and 100%. Muns${}^{3}$ in particular shows a first quartile at 90% accuracy, evidence that deepCC was seleting a Munsell chip within the direct vicinity of the correct one in the vast majority of cases under this condition. ΔE and CCI measures lead to the same conclusions: Median ΔE is found at 3.3 and a third quartile at 9.40, thus showing that in the large majority of cases, the model showed an error of the same magnitude as the interdistance between Munsell chips in CIEL*a*b*. The analysis of the CCI distribution leads to the same conclusions: We found a median value of 0.62 but a first quartile at –0.43. This indicates that, while for most images DeepCC performs relatively well under the no patch condition (a CCI of 0.62 remains in the upper-half of CCI values reported in humans psychophysics), it is generally more difficult for the model to solve the task, to the extent that a significant proportion of images yields a negative CCI.

Interestingly, the reliance on the back patches’ presence was not equal across DeepCC's training instances. One instance saw its accuracy change by merely 20%, while another experienced a drop of 60%. Refining our scene manipulations, we also looked at how the model's instances responded when masking one colored patch in the background at a time. Some patches appeared more critical than others: Masking the red and yellow patches (second and fifth from the left) led to the largest loss in accuracy, with average losses of 9.9% and 8.9%. Masking the white and black patches (third and sixth from the left), however, had the least impact on the model's performance, accounting for losses of 0.1% and 4%, respectively, on average. Individual differences were also confirmed. When masking the red patch, for example, one instance dropped by 22% in accuracy, while another dropped only by 2.3%. Some instances were also mainly affected by the red patch, others by the yellow patch. Nevertheless, the relatively high accuracies and CCI show that the model remained able to perform the task, albeit less successfully. The fact that different patches had different influences also tends to suggest that the decline in performance was not just a generic decline associated with deviations from the training set, but rather reflected the use of specific information from the patches to support color constancy.

These results are evidence that DeepCC indeed uses the information provided by the six constant colored patches in the back wall—particularly the chromatic ones. This is confirmed by the performance obtained for the model on the *wrong patch* dataset (data not shown). Indeed, we found that the models performed equally well or worse under this condition than under the *no patch* condition. Contrary to the latter, *wrong patch* introduces a conflicting cue rather than the absence of one, thus making the task even more difficult for the model if it partially relies on the colored patches in the background. Still, we found a median CCI value of 0.22, thus showing that despite the conflicting cues, the model retained some degree of color constancy and must rely on other cues to account for the illumination's color.

In the *wrong background* condition, however, DeepCC's performance dropped considerably, with a median top-1 accuracy at 0, and median CCI values below 0 for all training instances. In fact, its performance dropped to the same level as our control model's Deep65 tested on the same dataset. DeepCC shows a median ΔE error of 11.4, for instance, and 11.3 for Deep65. In the *wrong background* dataset, the background was manipulated such that it appeared constant across all test illuminations, and illuminated by our reference D65. This near equality is strong evidence that DeepCC relies solely on the contextual cues surrounding the floating object to perform color constancy: When deprived of these cues, it interprets any chromaticity shifts induced by the test illuminations as being intrinsic to the object's surface and thus wrongly classifies its color, just like Deep65 would.

### Interim conclusion

Thanks to the controlled manipulation of the scene surrounding the floating object, we saw in this section that all DeepCC instances solely rely on contextual cues to identify the object's Munsell surface and account for illumination change: When deprived of reliable cues surrounding the object of interest, it behaves the same as Deep65, the same architecture trained with the D65 illumination only. Similarly, humans rely on contextual cues to solve color constancy (Kraft & Brainard, 1999; Kraft et al., 2002). Individual differences were observed between training instances, however, when the colored patches in the background were removed, with some instances relying more on certain patches than others.

## Standard approaches

To further evaluate DeepCC, we compared its performance to the error expected with classical approaches to illumination estimation, coupled with the von Kries correction (Von Kries, 1902), standard in computer vision (Akbarinia & Parraga, 2017; Hu et al., 2017).

### Methods

For comparison purposes we also computed, on our test images of the CC normal condition, the errors expected from classical approaches to illumination estimation: *Gray World, White Patch* (Land, 1977), and *adaptive-surround modulation* (ASM) (Akbarinia & Parraga, 2017). All of these approaches are driven by low-level features (as opposed to learning): Gray World makes the assumption that the world is on average “gray” under a neutral illumination and takes the average pixel value as an estimation of the illumination's color; White Patch considers the brightest pixel as an estimation of the illumination; ASM assumes that image areas with high to middle spatial frequencies (typically edges) are most informative and computes the illumination by dynamically pooling a portion of the brightest pixels according the average image contrast. Each of these approaches delivers a single global triplet of values specifying the illuminant for a given image.

To enable a link from the global illumination estimations to our classification task of the floating object's surface color, we coupled these approaches with a global von Kries correction (von Kries, 1902). This correction consisted in dividing each image pixel by the three illumination values estimated by each approach. For each resulting image, we then segmented the floating object and estimated its chromaticity by considering the mean value of all its pixels. We then compared this estimated chromaticity with the chromaticity found for the exact same object, at the exact same position and orientation, but under a reference illumination. In this way, any difference between the estimated chromaticity and the reference chromaticity would be a consequence of the illumination estimation + correction only. As a reference, we used the computed chromaticity of the object rendered under the D65 illumination.

Of course, there are many other approaches to illumination estimation and white-balance correction than the ones tested here, some of which may be more accurate (see Akbarinia & Parraga, 2017, for a review; Shi et al., 2016; Afifi & Brown, 2019). All of them, however, deal with RGB images and rely on the global von Kries adaptation for the final correction, which in itself is an approximation. As an upper bound for any approach based on illumination estimation and von Kries adaptation, we also estimated the error of the von Kries method based on the ground truth illumination (perfect illumination estimator) using the same evaluation procedure as for estimated illuminations. This object color estimate is not perfect, because it does not take into account local variations in illumination, due to interreflections within the scene, for instance (Worthey & Brill, 1986; Flachot et al., 2016; Foster, 2011). Finally, we also computed the error obtained without compensating for the illumination at all. This would serve as an error estimate for a model that would perfectly segment the object of interest in the scene, but not compensate for the illumination (a perfect non–color constant model). By definition, such a model would thus have a CCI of 0.

### Results

Figure 3C shows the distributions of ΔE errors and CCI predicted from the classical approaches to color constancy, together with the results obtained under the *normal* and *wrong background* conditions, described previously, for comparison purposes.

We found median ΔE values for all of the aforementioned approaches to be higher than for DeepCC under the *normal* condition. Even the error merely induced by the von Kries adaptation (perfect illu condition in the figure) leads to higher errors, with a median value of 2.9. This median value is in fact very similar to the median found for the *no patch* condition, although slightly better. This is confirmed by the corresponding median CCI of 0.65. Of the classical approaches, the Gray World hypothesis proved to be the most accurate, with median values of 4.6 ΔE and 0.48 CCI, slightly worse than for DeepCC on the *no patch* condition. This suggests that not only did the DeepCC instances accurately identify the region of interest that is the object within the image and managed to accurately estimate the illumination, but they also accounted for the object's position with respect to the illumination. It also implies that DeepCC found a better correction strategy than a global discounting of the illumination like in the von Kries approach. This is presumably thanks to the nature of the task, which tries to estimate object color rather than a global illumination, and thanks to the convolutional nature of the model's architecture, which allows local discounts of the illumination.

Although DeepCC under the *wrong background* condition exhibits errors greater than every one of the standard approaches, it is as well to note that its distribution is quite close to the distribution predicted for a perfect non–color constant model (*no CC* condition in the figure). Indeed, we find a median error of 9.4 ΔE for the *no CC* condition, similar to the 11.4 ΔE found for the *wrong background* condition. This suggests that DeepCC is indeed misled to attribute a neutral illumination on a floating object and thus behaves like a non–color constant model. Since Deep65 performs at the same level as DeepCC on the same dataset, it is likely that the discrepancy of 2 ΔE between *no CC* and *wrong background* comes from the fact that DeepCC is no perfect Munsell classifier, even with all contextual cues available.

Gray World's success compared to other approaches can be explained by the relative simplicity of the scene: a room with fairly neutral walls, with a single illumination. ASM would be expected to perform better using images of more complex scenes. The poor performance of the White Patch approach for many images can be understood by the proximity of the object of interest to the camera: When a Munsell reflectance of high value is applied to the object, the brightest pixels are likely to be found on the object itself, rather than on some other parts of the context.

### Interim conclusion

Comparisons with classical approaches to color constancy show that under the *normal* condition, DeepCC learned how to compensate for the illumination better than any of the classical approaches we tested. It even performed better than a hypothetical model provided with omniscient knowledge of the true illumination and compensating through the von Kries correction, the standard procedure for discounting in a scene the illumination after its estimation (Akbarinia & Parraga, 2017). Under the *wrong background* condition, its performance lies close to the predicted performance of a model that would perfectly segment the object of interest in the scene and extract its chromaticity, but not account for the illumination color. This suggests that similarly to humans, it also relies on context to achieve color constancy (Kraft & Brainard, 1999; Kraft et al., 2002; Yang & Maloney, 2001).

## Effect of illumination

To test the DeepCC models, we used the four illuminations: Yellow (Y), Blue (B), Green (G), and Red (R) (see Figure 1C). These were chosen to be equidistant to D65 in CIEL*a*b* space, with Y and B on the daylight locus and G and R in the orthogonal direction. Note, however, that even though none of these four illuminations were used during training, Y and B are expected to appear more “familiar” to the models than the other two. Indeed, the distribution of natural illuminations used for training includes several other illuminations along the daylight locus. G and R, however, were outside the distribution of the training set. More precisely, G is 4.45 ΔE away from its closest illumination within the training set, while R is 7.9 ΔE away, making R then G the two illuminations DeepCC is less familiar with.

**Figure 5.**
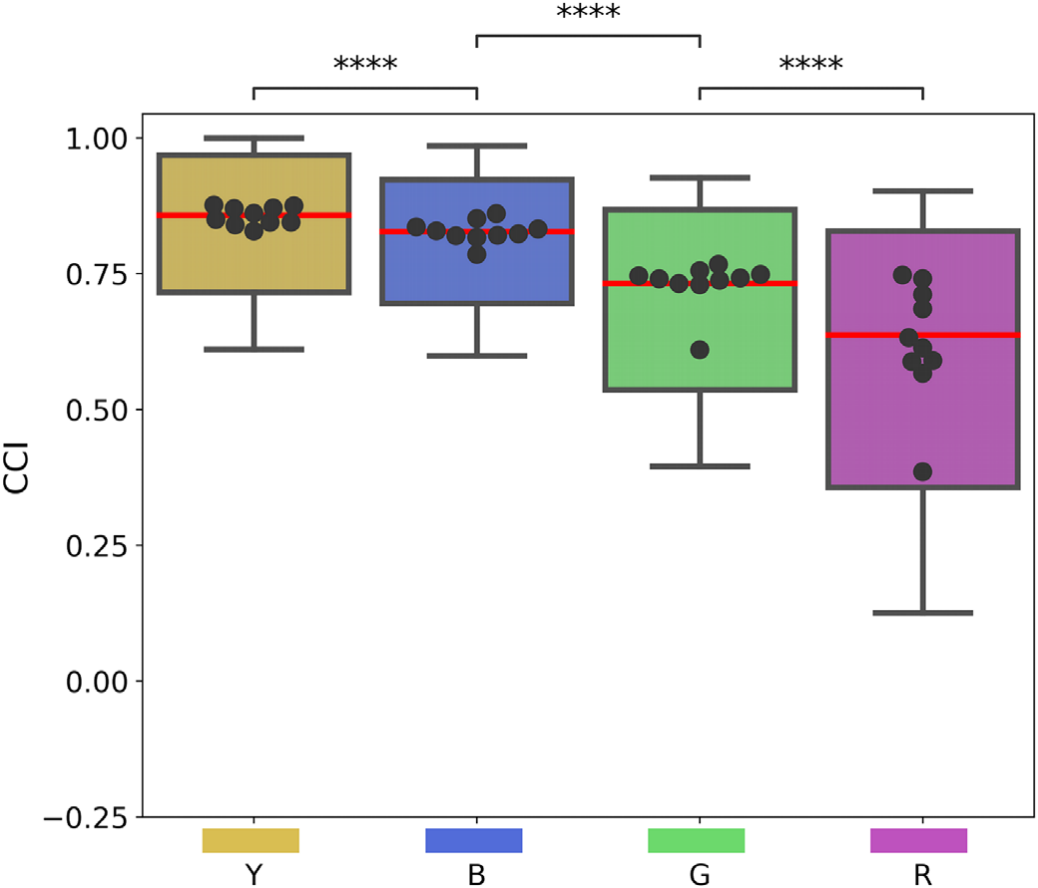
Effect of the illumination on color constancy: distributions of DeepCC's mean Color Constancy Index (CCI) for each Munsell class under each of the four testing illuminations. Medians are in red. Each dot of the bee swarm plots is to the average CCI found for a training instance of DeepCC. Statistical significance was computed applying pairwise *t* tests with Bonferroni corrections.

This anisotropy in the distribution of natural illuminations had consequences on the performance of our models and their degree of color constancy. For each training instance and illumination, we computed the mean CCI per Munsell class and training instances, with each mean value computed across 10 image exemplars in the *normal* conditions. Figure 5 shows the distributions of these mean values for each of the four illuminations in the form of a boxplot. Additionally, we also plotted the average CCI value for each training instance under each illumination in the form of bee swarms. We observed a significant effect of the illumination on the CCI of our models: DeepCC models showed higher CCI for the “familiar” illuminations (Yellow and Blue) than for the “unfamiliar” illuminations (Green and Red). The highest degree of color constancy was found under the Yellow illumination, with an average CCI value of 0.86, while the lowest was found under the Red illumination, with an average CCI value of 0.64.

Results of Figure 5 are very similar to observations made regarding the capacity of humans to perceive illumination changes (Pearce et al., 2014; Aston et al., 2019). It was found that human observers were more sensitive to illumination changes happening along the green–red color direction compared to changes along the yellow–blue direction, meaning that they are less likely to perceive an illumination shift along the yellow–blue direction than along the green–red one. This suggests, the authors argue, that the human visual system compensates better for changes in the blue–yellow directions, which could have consequences for color constancy.

### Interim conclusion

Results in this section show a significant effect of the illumination on DeepCC's performance. Higher color constancy indices were observed for illuminations along the yellow–blue direction in CIEL*a*b* color space compared to illuminations falling onto the orthogonal direction. This difference is presumably explained by the model being more accustomed to variations along the daylight locus, the direction along which daylight and natural illuminations, such as the ones used for training, vary most. The parallel one can draw between our result and observations made in human psychophysics (Aston et al., 2019) implies that the higher variation along the daylight locus may be a cause of similar consequences in humans.

## Color constancy throughout DeepCC

There is uncertainty regarding where the neural mechanisms for color constancy would take place in the brain. Many studies emphasize early mechanisms, such as cone adaptation (Lee et al., 1999), or cells sensitive to chromatic contrasts between object and background in V1 (Wachtler et al., 2003). Other have shown that lesions in macaque area V4 also led to impaired color constancy (Wild et al., 1985; see Foster, 2011, for a review). In contrast to biological brains, deep neural networks like DeepCC allow access to the activations of every unit. Taking advantage of this, we added linear readouts to every layer of DeepCC in order to measure at which processing step color constancy emerges.

### Methods

In order to apply the Color Constancy Index at different processing stages of DeepCC, we trained readout networks for each one of its five layers (three convolutional and two fully connected). These linear probes (Alain & Bengio, 2016) consisted of very simple, fully connected linear models with 1,600 kernels, 1 per Munsell class. They take as input the ReLU-corrected output of DeepCC's layer they read out, before the maxpooling operation. For example, the readout network of DeepCC's first convolutional layer (RC1) takes as input the output of that layer after the ReLU operation and is trained on the same task as DeepCC, using the same dataset. The parameters of DeepCC's convolutional layer are not updated during this training iteration, only the weights of RC1. RC1 being fully connected and linear, no complex or nonlinear operations are added, and as such, RC1's performance is an indication of the amount of information available in the first convolutional layer of DeepCC.

### Results

**Figure 6.**
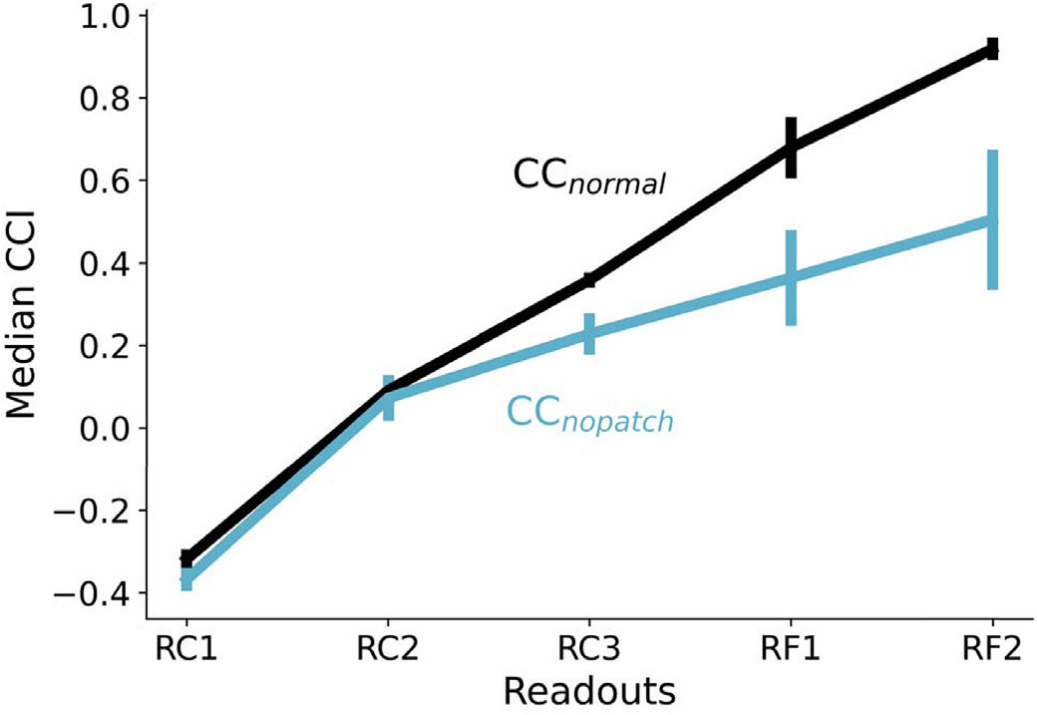
Color Constancy Index (CCI) for the five readout models tested with the *normal* and *no patch* image sets. Each readout takes input from all units of the designated layer: from the three convolutional layers (readouts RC1, RC2, and RC3) to the two fully connected layers (readouts RF1 and RF2). By extension, the value of CCI reflects the degree of color constancy at the different layers of DeepCC.

Figure 6 shows the average CCI obtained for DeepCC readout models. We named these readout models RC1, RC2, RC3, and RF1, RF2, corresponding to the convolutional layers 1, 2, 3, and the fully connected layers 1, 2, respectively. We trained 10 instances of each readout model, one for each instance of the original model. As shown in the plot, the readout models were tested under two conditions: CC${}_{normal}$ (black) and CC${}_{nopatch}$ (cyan). Error bars are the standard deviation obtained across the 10 training instances. The CCI gradually increases in the normal condition in an almost linear fashion across processing stages, consistently across the 10 models. In the *no patch* condition, CCI follows the normal condition only up to RC2, at which point it continues increasing but at a much lower rate. The difference between the two conditions becomes significant from RC3 onward. Error bars are also larger for the following layers, another indication of the large individual differences between training instances and observed in “Impoverished visual scene”.

### Interim conclusion

Contrary to many physiological studies emphasizing the early neural mechanisms for color constancy (Foster, 2011), we found that color constancy seemed to increase steadily throughout DeepCC, both under the normal condition and the no patch condition.

## Color representations in DeepCC

We next performed a representational similarity analysis (Kriegeskorte et al., 2008) on unit activations within each layer to probe the models’ internal representations of colors. We find that although the training objective treated each Munsell value as an entirely distinct class, the DeepCC networks nonetheless learned similarity relationships between the colors that closely resemble their true embeddings in the Munsell space.

### Methods

To estimate the similarity between Munsell colors as seen by DeepCC, we computed representational dissimilarity matrices (RDMs) (Kriegeskorte et al., 2008) between the average unit activations per Munsell classes for each layer in the DeepCC networks using the correlation distance as a metric (Aguirre, 2007). Activations were recorded using the evaluation dataset under the normal condition, augmented with additional images under the D65 illumination (i.e., the 330 test Munsell classes under the D65, Y, B, G, and R illuminations). In turn, the RDMs were used as input to a classical multidimensional scaling analysis (MDS) (Cox & Cox, 2008) to compute the underlying dimensions best explaining the previously found dissimilarities. Previous work has shown that the activations of complex deep neural models were able to predict neural response in biological brains (e.g., in mice), even when untrained, that is, with random weights (Cadena et al., 2019). As a control, we thus also performed the same analysis for 10 instances of the deep architecture with random weights, denoted *DeepRand*.

### Results

**Figure 7.**
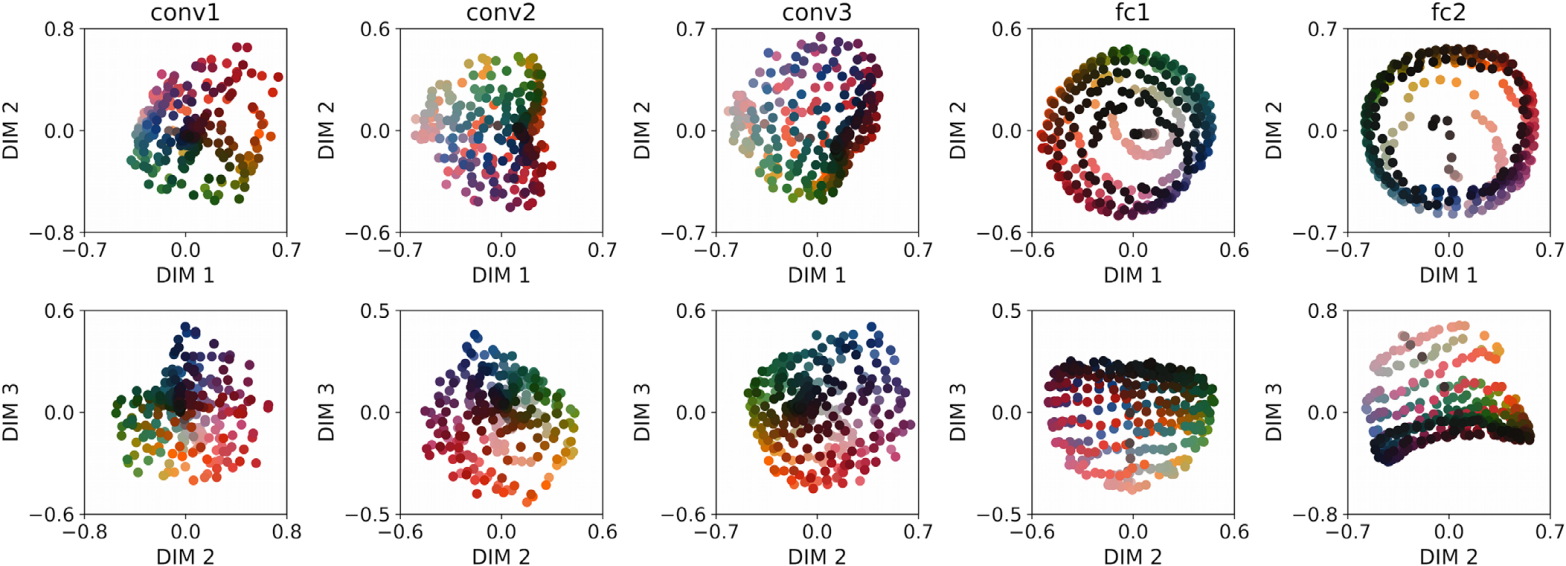
Results of a multidimensional scaling performed on the correlation of Munsell representations for different layers of DeepCC, from convolutional layer 1 (Conv1) to fully connected layer 2 (Fc2). Each column corresponds to one layer, each row to the different dimensions resulting from the MDS: first row, Dimensions 1 and 2 of maximal variance (decreasing order); second row, Dimensions 2 and 3 of maximal variance (decreasing order). Each dot corresponds to one Munsell surface, displayed with its color under the D65 illumination. While Munsell surfaces appear clustered in the early layers, particularly with respect to lightness, a progressive disentanglement in terms of chromaticity and lightness takes place throughout the network.

We performed MDS on the RDMs for each of the five layers of DeepCC. Figure 7 shows two-dimensional (2D) representations of the first three dimensions of the MDS results for each layer, tested under the *normal* condition and averaged across all 10 training instances. These three dimensions are the dimensions of maximal variance, in decreasing order. Each column corresponds to one layer. The upper row plots the first and second dimensions, the lower row the second and third. Colored dots correspond to Munsell chips and are displayed using their corresponding sRGB values.

We find that increasingly human-like color dimensions emerge in all layers: Munsells are separated according to their lightness, sometimes also their hue. There is a progression in the way DeepCC represents Munsells: In early layers, many colors are clustered together, especially in the dark regions, rendering them less easily discriminable from one another. This changes in the last two layers, in which colors are more clearly separated from one another. Additionally, the dimensions are easy to interpret. In the first fully connected layer, for example, each dimension seems to code for a standard color dimension: Dimensions 1 and 2 for “yellow–blue” and “red–green,” with an almost perfect hue color circle and a radius correlated with saturation, and dimension 3 for lightness.

At each layer, we also computed the cumulative percentage of activation's variance explained by the three first dimensions given by the MDS, both for DeepCC and DeepRand, the latter consisting of deep instances with random weights. We interestingly found that, although the MDS could potentially yield a much larger number of dimensions, the first three dimensions are enough to explain more than 85% of the variance in most of the layers, for both model types. The highest percentage of explained variance in DeepCC is found for fc1, with 91%. This means that the representations of Munsell are mostly 3D. This result is particularly surprising because fc1 contains the highest number of kernels (250, same as fc2) and thus is more likely to lead to a higher-dimensional latent space. And indeed, the explained variance is lowest at fc1 layers for DeepRand, with 68%.

**Figure 8.**
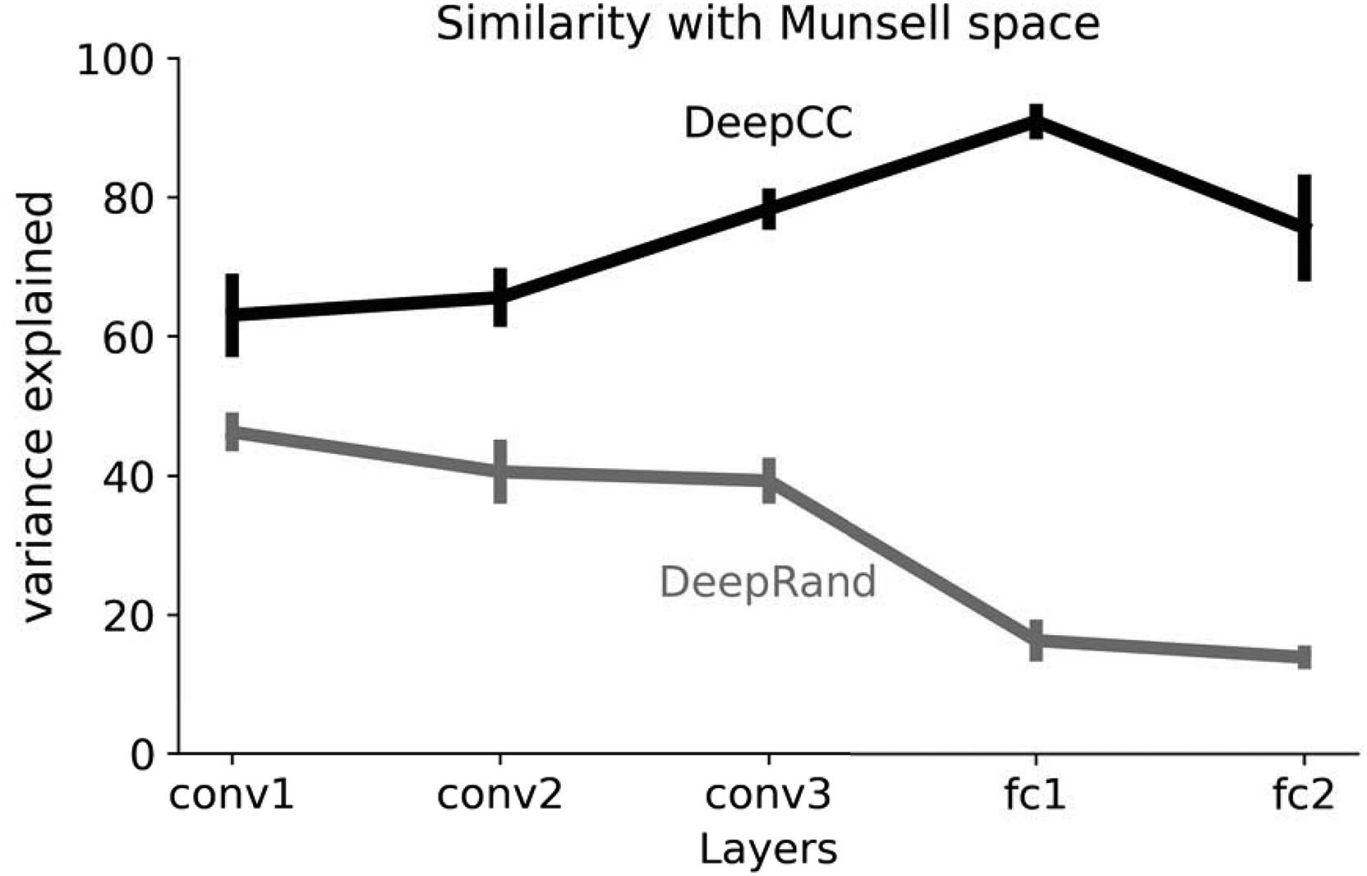
Result of the similarity analysis for all layers of the deep architecture trained on the CC dataset (DeepCC) and with random weights (DeepRand), from convolutional layer 1 (conv1) to fully connected layer 2 (fc2). The figure shows that the highest similarity with Munsell coordinates was found for DeepCC at the first fully connected layer fc1. Additionally, DeepCC always rates higher than DeepRand.

We next sought to quantify the similarity—or difference—between Munsell representation in our models and their coordinates in a perceptual color space. To do this, we performed a Procrustes analysis (Gower, 1975) to identify the rigid transformation that best mapped the coordinates obtained from the first three MDS dimensions, performed on each layer, to the corresponding coordinates in the Munsell color space. The percentage of explained variance is an indication of the goodness of the mapping: The closer to 100%, the better. As shown in Figure 8, we find that in all layers, the variance explained by DeepCC progressively increases from 63% in convolutional layer 1 to 91% in fc1. Fc2's subsequent drop likely reflects the demands of the objective function to deliver categorical outputs. Additionally, DeepCC significantly explains more of the variance than the same architecture with random weights (DeepRand) with a maximal difference in fc1. Indeed, while the variance explained progressively increases for DeepCC, it progressively decreases for DeepRand. Note the relatively high explained variance for both DeepCC and DeepRand models in the first layer conv1. It is likely a consequence of the input space: Performing the Procrustes analysis from the Munsell chromaticities in LMS space (input) to Munsell coordinates yields a percentage of accounted variance of 66%, very close to the 63% found in DeepCC's conv1.

It is important to note that this finding is nontrivial and cannot be explained solely by the loss function we used. During training, the networks were never taught similarity relationships between Munsell color values. Rather, the error signal was the same whether the models wrongly selected a Munsell close to or far from the correct one in color space. Theoretically, a model could reach a high accuracy and not learn human-like similarities between the Munsell colors. And indeed, as reported below, other architectures trained and tested following the same procedures represent colors in a different manner.

Qualitatively similar results were also obtained when using a L2 norm instead of the correlation metric. Additionally, we also performed this analysis using the CIEL*a*b* coordinates as a reference for the Procrustes analysis and found extremely similar results as with the Munsell coordinates. We excluded these results from the figures to avoid redundancy.

### Interim conclusion

Similarly to the increasing CCI observed throughout the network in the previous section, the representational analysis also uncovered a progression in the way Munsell colors are represented within the model's layers. Visually, we could observe a progressive disentanglement of Munsell colors with increasing layer depth. More important, the representation of color also progressively increased their resemblance with human perception, peaking at FC1, where there was a very high correspondence to the Munsell perceptual color space. This was quantitatively confirmed using a similarity analysis, where it was found that the representational distances and dimensions between Munsell values, in the penultimate layer in particular, matched very well the human perceptual distances and dimensions found empirically in previous psychophysical studies. The subsequent drop found in the last layer likely reflects the demands of the objective function to deliver categorical outputs.

## Standard and custom architectures

We observed in the previous section that DeepCC represents Munsell colors following color dimensions found empirically to be perceptually relevant for humans. Is this a special feature of this architecture (i.e., would different architectures learn different representations)? If yes, it would be strong evidence that there is not one globally optimal system of representations to solve color classification. To answer this question, we trained and evaluated several other standard deep learning architectures.

### Methods

#### Architectures

For the sake of comparison, we also trained three standard, high-performance deep learning models on the *CC* dataset: VGG-11 (Simonyan & Zisserman, 2014), MobileNet (Howard et al., 2017), and ResNet-50 (He et al., 2016). All of these architectures have specific features that make them significantly different from one another. These standard architectures, however, are relatively large and complex compared to the DeepCC architecture. While DeepCC only has 676 kernels (outside of the classification layer's 1,600) and 3.6 million interconnections between units, all three others have more than 13,000 kernels, the highest being ResNet-50 with almost 54,000. In order to allow some comparison with networks of a size more similar to DeepCC, we additionally devised another, shallower model. It consisted of a custom ResNet architecture, generated thanks to a ResNet bottleneck architecture generator (available in github^7^). To distinguish it from ResNet-50, we will call this architecture *ResCC*. It has three layers, each with three, one, and two bottleneck blocks, respectively. The first layer starts with 16 kernels, layer 2 with 32, and layer 3 with 64. Including the kernels within the bottleneck layers, it reaches 3,424 kernels and 0.6 million interconnections. Similarly to DeepCC, where we trained 10 instances, 6 independent training instances of ResCC were trained for further analysis.

### Results

We evaluated each one of the DNN architectures on the *normal*, *no patch*, *wrong patch*, and *wrong back* conditions. Here, for the sake of simplicity, we show only a summary of the results through a table with the distributions’ medians. Table 1 shows the median measurements of performance obtained for all architectures under those conditions, with the results obtained for DeepCC as a reminder on the last column. MobileNet, VGG-net, ResNet-50, and ResCC all showed higher performance than DeepCC in all conditions. Interestingly, there was almost no difference in performance for every model other than DeepCC between the *normal*, *no patch*, and *wrong patch* conditions. All models, however, have shown a significant loss in accuracy for the *wrong back* condition, suggesting that all tested models rely heavily on cues in the background to perform their task.

**Table 1.** Median values found for all measures and all models under the normal, no patch, wrong patch, and wrong back conditions. All models show higher performances than DeepCC in all test sets. Interestingly, except DeepCC, none of the models are sensitive to the absence (*no patch*) or incongruence (*wrong patch*) of the colored patches in the background. This suggests that in contrast to DeepCC, these other models barely rely on the constant colored patches in the background to perform color constancy. The sharp drop in performance for the *wrong back* condition, however, suggests that like DeepCC, all other models also rely on the contextual cues surrounding the floating object to perform color constancy.

Model	MobileNet		VGG-11		ResNet-50		ResCC		DeepCC (ref ConvNet)	
Nb param	4.3 M		135.3 M		29.8 M		0.6 M		3.6 M	
Condition	*normal*	*no patch*	*normal*	*no patch*	*normal*	*no patch*	*normal*	*no patch*	*normal*	*no patch*
Top-1	95	95	100	100	100	95	85	80	75	40
Top-5	100	100	100	100	100	100	100	100	100	90
Muns${}^{3}$	100	100	100	100	100	100	100	100	100	95
Δ E	0.0	0.0	0.0	0.0	0.0	0.0	0.0	0.0	0.0	3.3
CCI	1.0	1.0	1.0	1.0	1.0	1.0	1.0	1.0	1.0	0.6

Condition	*wrg patch*	*wrg back*	*wrg patch*	*wrg back*	*wrg patch*	*wrg back*	*wrg patch*	*wrg back*	*wrg patch*	*wrg back*

Top-1	85	0.0	92.5	0.0	87.5	0.0	80	0.0	25	0.0
Top-5	100	25	100	25	100	25	100	25	65	25
Muns${}^{3}$	100	70	100	75	100	70	100	65	85	65
Δ E	0.0	10.6	0.0	10.0	0.0	10.34	0.0	11.2	5.6	11.4
CCI	1	−0.34	1.0	−0.28	1	−0.32	1	−0.4	0.23	−0.46

Up to now, standard networks and ResCC essentially shared the same characteristics as DeepCC: While they outperformed the classical approaches to color constancy, such as Gray World (cf. “Comparison with classical approaches”) under the *normal* condition, they failed to account for the illumination color under the *wrong back* condition (cf. Figure 3), as indeed essentially any observer would. Additionally, we found they also show a significant effect of the illumination on the Color Constancy Index, with higher performance for the Yellow and Blue illuminations than for the Green and Red illuminations (not shown).

However, when it came to the analysis of Munsell representations within the latent layers, they all exhibited a very different picture from DeepCC: Munsell chips did not appear to be differentiated following human-like color dimensions. As in the previous section, we performed multidimensional scaling on the RDMs for each layer of each architecture, followed by a Procrustes analysis using Munsell coordinates as a reference space. Across all architectures, the highest percentage of explained variance resulting from the Procrustes analysis was 53%. It was obtained for the VGG-11 architecture's fourth layer and stands substantially below the 91% explained variance of DeepCC's penultimate layer.

**Figure 9.**
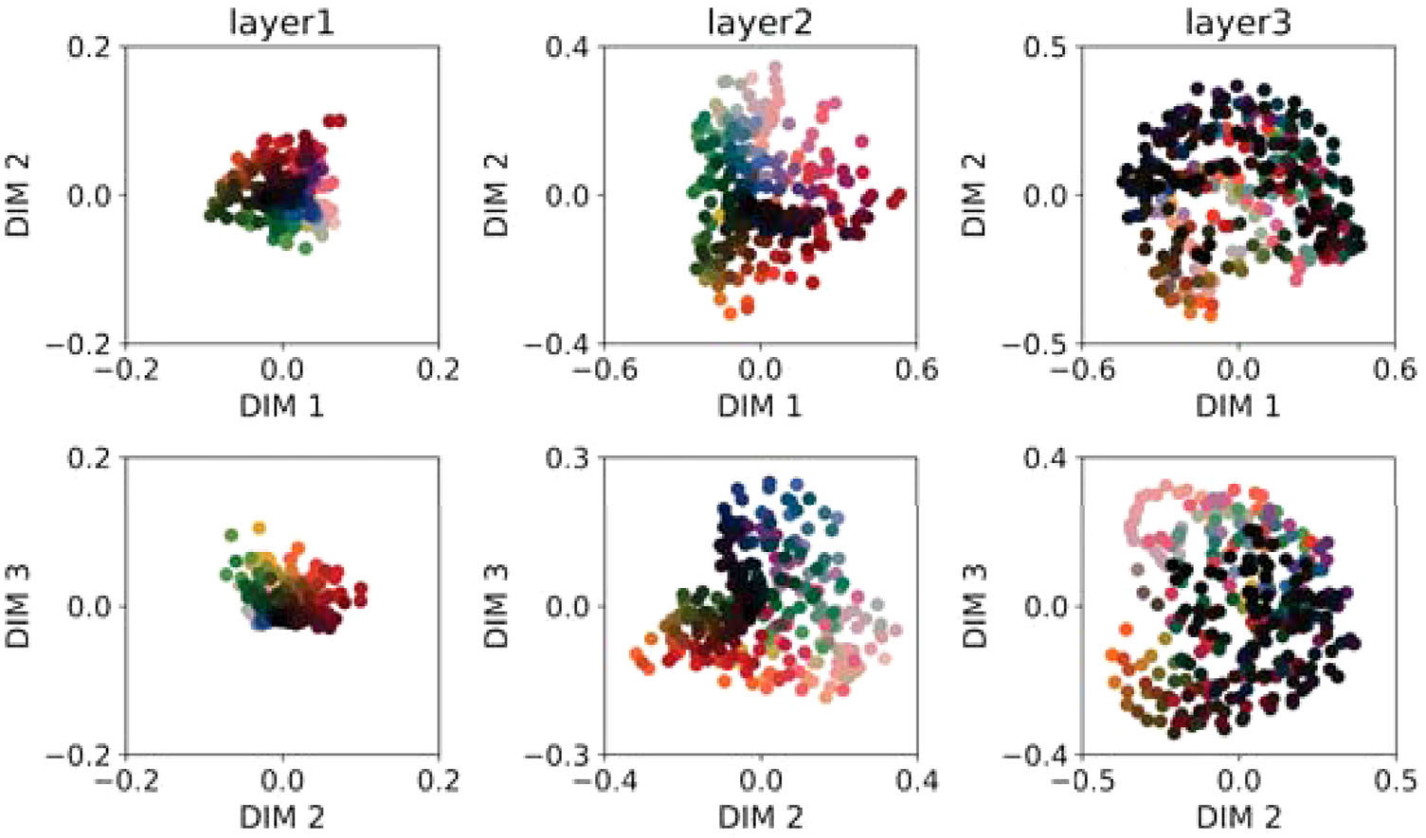
Results of a multidimensional scaling performed on the correlation distance of Munsell representations for different layers of ResCC. Compared to DeepCC (cf. Figure 7), ResCC does not seem to classify Munsells following the same dimensions as those defined by human perception, particularly in Layer 3.

As an example, we show in Figure 9 the results of the MDS analysis averaged over the ResCC instances. We can observe that none of the three layers visibly separate Munsell colors along human-like perceptual dimensions like Hue, Lightness, or Chroma. This is particularly true for Layer 3. For this last layer, the first three dimensions of the MDS account for only 54% of the dissimilarity between Munsell representations, meaning that Munsell discrimination took place in a space with more than three dimensions.

**Figure 10.**
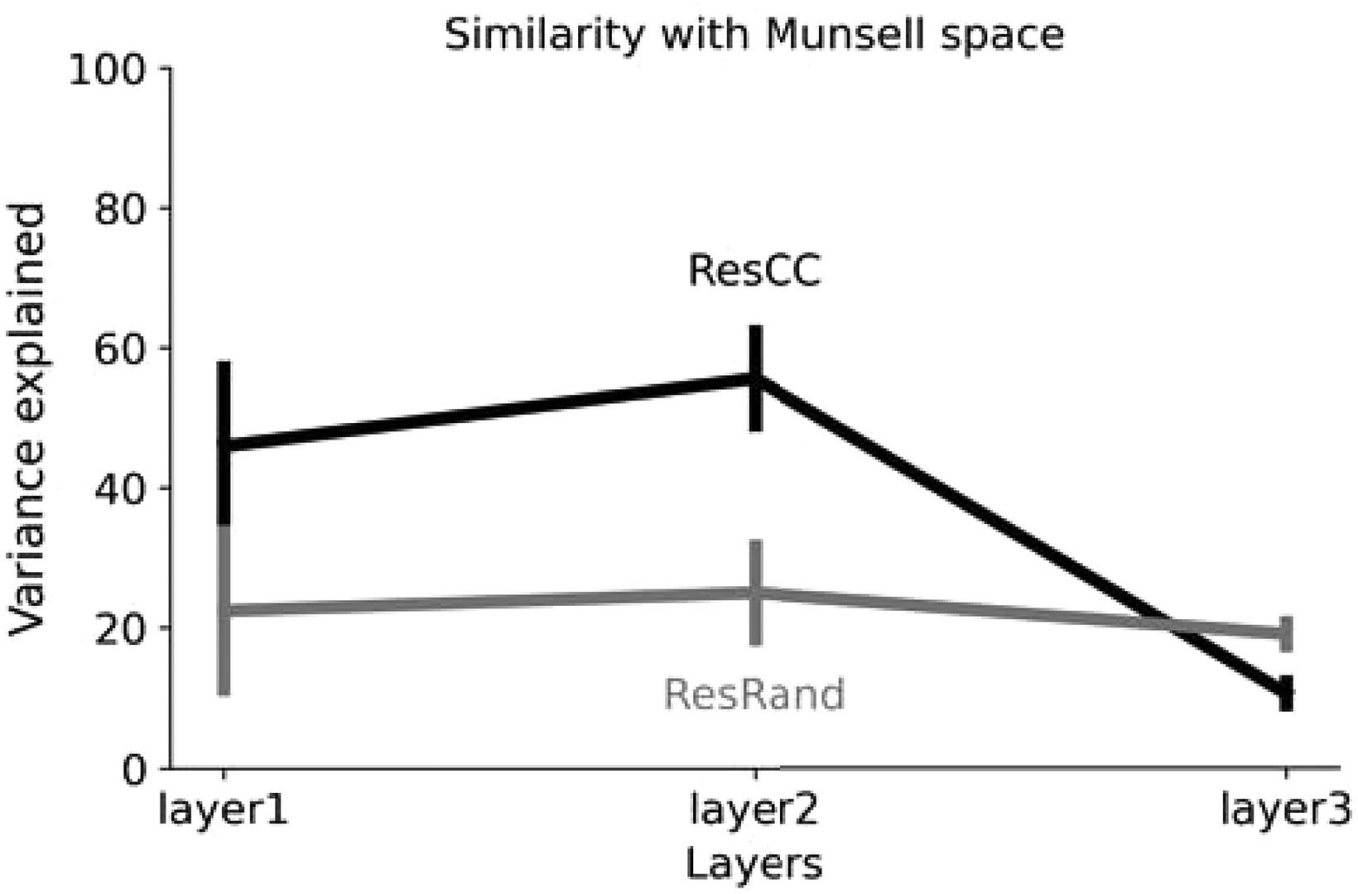
Results of the Procrustes analysis for the Res architecture trained on the CC dataset (black) and with random weights (gray). The analysis was performed on the outcomes of the multidimensional scaling at different layers using Munsell space as reference coordinates. The variance explained for ResCC was consistently lower than for DeepCC throughout its layers, meaning that ResCC discriminate Munsells following color dimensions dissimilar to those defined by human color perception. The fact that the Res architectures systematically rate lower both when trained and with random weights suggests that the major factor for this difference is the architecture.

This observation is further confirmed by Figure 10. The variance explained by the best fit for mapping Munsell representations in ResCC layers onto the Munsell coordinates was always lower than for DeepCC, meaning that ResCC distinguished Munsell values using color dimensions different from the ones defined by human color perception, contrary to DeepCC. Additionally, the low percentage of variance explained by the same architecture but with random weights (ResRand) suggests *that the architecture is the major factor for this difference*. Interestingly, this result correlates with a recent observation (Zhou et al., 2018) that ResNet architectures lead to much less interpretable representations than more conventional convolutional architectures like AlexNet and VGG Nets.

### Interim conclusion

The results of our comparisons with other architectures show that if performance was our only goal, many architectures other than *Deep* could have been used to solve the Munsell classification task and indeed achieved superior performance. The similarity analysis we used, however, showed that other architectures, such as ResCC, seemingly differentiate between Munsell colors according to color dimensions very different from those empirically found for human perception, contrary to DeepCC.

This last observation is thus evidence that there is not one globally optimal system of representations to which all networks tend to converge. Rather, multiple possible systems of representations deliver good performance at the task, the one shared between humans and DeepCC being one of them. This result also emphasizes the need for careful examination when it comes to selecting a DNN architecture for a given task. While at first sight, ResCC might have seemed a better choice for our tasks (highest performance and few parameters), the analysis of the Munsell representations shows that DeepCC presents characteristics more similar to human color discrimination. This last point suggests that DeepCC is thus potentially a better candidate for modeling human discrimination of Munsell color surfaces. It also emphasizes the need to develop further methods and strategies to analyze and understand the features learned by different architectures.

## Discussion

We have trained deep neural models for the classification of Munsell chips under varying natural illuminations using 3D spectral renderings. We found that our models did learn to discount the illumination's contribution to the color appearance of the surface, hence learning color constancy. When manipulating the contextual cues within the scene, in such the way that these cues no longer gave information about the illumination shining on the object, our models were no longer color constant, performing exactly at the same level as our control network Deep65, trained under our reference illumination D65 only. Additionally, we found that despite using the same training procedure, different architectures led to very different color representations of Munsell chips within their layers: One network, DeepCC, developed color representations very similar to the Munsell chips coordinates, while the other models did not.

In the following, we discuss how these findings relate to human color constancy and color vision. We also discuss the opportunities offered by the combination of deep learning and computer graphics for studying properties of human vision such as color constancy.

### Deep neural networks for biological color vision

We find that as a result of training, the deep neural network models became similar to humans in several respects: They classified Munsell colors largely independently of changes in illumination, thus learning color color constancy. They used contextual information to do so: When we manipulate the scene elements to provide incorrect information about the illuminant, the models perform at the same level as a non–color constant model, meaning that they are no longer able to discount the illuminant. Likewise, numerous previous studies have shown that humans also rely on context to achieve color constancy (Kraft & Brainard, 1999; Kraft et al., 2002; Yang & Maloney, 2001). One model, DeepCC, was also sensitive to the cues provided by the constant color patches in the background. Additionally, the models showed higher degrees of color constancy for illuminations along the daylight locus than for illuminations along the orthogonal color direction. This also correlates with the lower sensitivity to illuminant change along the daylight locus observed in humans (Aston et al., 2019).

In addition, our analysis of the networks’ inner representations revealed that DeepCC represented surface colors using dimensions similar to the Munsell and CIELab spaces, which are based on human perception. This similarity seems to be the exception rather than the rule, as other architectures like ResCC, represented color in a different way, despite achieving similar or superior performance on the objective. The observation that one architecture learned human-like features and not the other hints at architectural influences shaping human color perception. Better understanding these architectural influences—and how they relate to the architecture of primate visual systems—may help us understand human color vision in the future.

It remains unclear what exact mechanisms within the networks are responsible for achieving color constancy, and to what extent these are comparable to neural mechanisms found in biological visual systems. Some possibilities, however, are more likely than others. One mechanism thought to significantly contribute to primate color constancy is *adaptation* (Foster, 2011) present as early as at the retinal level (Lee et al., 1999). Adaptation, however, is commonly accepted to require either neural feedback from recurrent interactions within the network (del Mar Quiroga et al., 2016), or an intrinsic suppression mechanism in the neuron itself (Whitmire & Stanley, 2016), neither of which are explicitly implemented in the architectures used here: They are feedforward networks with simple ReLU activation functions. Recently, Vinken et al. have implemented an exponentially decaying intrinsic adaptation state within each unit of a feedforward CNN architecture (Vinken et al., 2020). They were successfully able to reproduce neurophysiological and perceptual properties of adaptation. Their proposed architecture could thus have the potential to learn the adaptation mechanism for color constancy if trained on our task. Nevertheless, the fact that networks can achieve color constancy without such adaptation mechanisms suggests that in humans, the primary role of adaptation may be in controlling sensitivity given limited dynamic range and noise, rather than surface reflectance estimation per se. Another mechanism thought to contribute to color constancy in biological brains is *cell response invariance*, or the tendency of certain cells to be largely sensitive to chromatic contrasts between target and background (Foster, 2011), both at the early stages of the visual system (Wachtler et al., 2003) and the later stages (Kusunoki et al., 2006). Recent studies have shown that kernels sensitive to chromatic contrasts can be found in the early and late convolutional layers of feedforward CNNs trained for object recognition (Flachot & Gegenfurtner, 2018, 2021; Harris et al., 2019).

### 3D-rendered dataset for color constancy

Unfortunately, large datasets consisting of numerous photographs of real, complex scenes with controlled conditions suitable for training deep neural networks from scratch on color constancy tasks do not yet exist. The popular ImageNet (Deng et al., 2009), for instance, consists of millions of natural images but taken from noncalibrated cameras, presumably white-balanced. The ColorChecker dataset (Gehler et al., 2008) has the opposite characteristic: It presents precise and well-calibrated complex images, but less than 1,000 of them. Large hyperspectral datasets of natural scenes at different times of the day would be optimal, of course, but the difficulty of controlled hyperspectral captures is such that most datasets count a few hundreds of images at most (Vazquez-Corral et al., 2009; Nascimento et al., 2016).

Some challenges remain, however, such as the efficient creation of convincing outdoor scenes. It is possible that reproducing the statistics of more complex, naturalistic scenes would contribute toward greater robustness of DNNs to scene changes and perhaps allow the emergence of higher features of color vision, such as color categories (Witzel & Gegenfurtner, 2018; Parraga & Akbarinia, 2016).

### Implications for color constancy in general

Our results have several implications for color constancy in general, independent of whether we believe that DNNs are a good model of human color constancy. First, we trained networks to extract the surface color more accurately than a perfect global von Kries correction. This implies that a global illumination correction is not the optimal solution to the color constancy problem, even in a situation with a single illumination color. This may guide future computer vision and image-processing work that aims to extract object properties rather than color-correcting images. Second, we confirm earlier suspicions that the prior distribution over illuminations causes the better performance of humans along the daylight axis, as employing a naturalistic range of illuminations was sufficient to cause our networks to have this bias as well. Third, our finding that network architectures like ResCC can achieve outstanding color constancy performance despite not reproducing human perceptual color similarity representations suggests that these representations are not necessary for color constancy. Although perceptual color spaces presumably have many advantages for human color vision, our findings do not support the notion that they are specifically optimized for color constancy—at least in the class of images we investigated. An interesting direction for future research would be to train networks explicitly on perceptual color representations and test how this improves performance at other tasks. This would potentially provide answers to the teleological question of why human color space is shaped as it is (DiCarlo et al., 2012).

## Conclusion

In this study, we approached color constancy as a surface reflectance classification task under varying illumination using deep neural networks. This methodology closely mimics what humans do on a daily basis and differs from the common approach to computational modeling of color constancy that mainly focuses on the illumination estimation and image correction. We then devised a set of testing conditions to thoroughly evaluate our models and compare them to previous human behavioural studies. We found that similarly to humans, all models heavily relied on contextual cues to solve color constancy and show the same bias toward illuminations along the daylight locus as humans. However, a similarity analysis on the activation patterns within the deep latent layers of the trained models showed significant differences in the way they represented color surfaces. Only one convolutional network, DeepCC, learned to discriminate colored surfaces following similar dimensions to those used by humans. This suggests that in computational models of human color constancy, the highest performance alone might not be the best metric to measure fidelity of a model to human color representations. This is in line with reports in object classification, where lower performance networks may better correlate with human brain recordings and behavioral measurements (Kubilius et al., 2019; Geirhos et al., 2020b).

## Footnotes

1 See https://colorconstancy.com/evaluation/datasets/ for a review.

2
http://www.mitsuba-renderer.org/.

3
http://www.cs.joensuu.fi/~spectral/databases/download/munsell_spec_glossy_all.htm.

4
https://evermotion.org/shop.

5
https://www.dropbox.com/sh/gz52alcoue9ew6w/AADYg3EJZD9bRLb04aifByNJa?dl=0.

6
https://github.com/AlbanFlachot/color_constancy.

7
https://github.com/ArashAkbarinia/kernelphysiology.
